# Evaluation of a Novel Dog Adoption Program in Two US Communities

**DOI:** 10.1371/journal.pone.0091959

**Published:** 2014-03-24

**Authors:** Heather Mohan-Gibbons, Emily Weiss, Laurie Garrison, Meg Allison

**Affiliations:** 1 Shelter Research and Development, Community Outreach, American Society for the Prevention of Cruelty to Animals (ASPCA®), Ojai, California, United States of America; 2 Shelter Research and Development, Community Outreach, American Society for the Prevention of Cruelty to Animals (ASPCA®), Palm City, Florida, United States of America; 3 Shelter Research and Development, Community Outreach, American Society for the Prevention of Cruelty to Animals (ASPCA®) Little Silver, New Jersey, United States of America; 4 Shelter Research and Development, Community Outreach, American Society for the Prevention of Cruelty to Animals (ASPCA®), New Orleans, Louisiana, United States of America; Knox College, United States of America

## Abstract

Millions of dogs enter animal welfare organizations every year and only a fraction of them are adopted. Despite the most recent American Pet Products Association (APPA) data that nearly half the US population owns a dog, only 20% acquired their dog from an animal welfare organization. Studies show that people consider adopting from an animal shelter more often than they actually do, which indicates a potential market increase if programs can make shelter dogs more visible to adopters. This research focused on a novel adoption program where shelter dogs were transferred into foster homes who were tasked with finding an adopter. Shelter dogs were placed in the path of potential adopters and bypassed the need for the adopter to go to the shelter. The results show that this novel program was effective in a variety of ways including getting dogs adopted. Although length of stay was significantly longer for dogs in the program, the dogs were in a home environment, not taking up kennel space in the shelter. The program also had a lower rate of returns than dogs adopted at the shelter. The foster program tapped adopters in different geographical segments of the community than the dogs adopted from the shelter. By bringing shelter dogs to where adopters spend their time (ex: restaurants, parks, hair salons), the program potentially captured a segment of the population who might have obtained their dog from other sources besides the shelter (such as breeders or pet stores). This novel approach can be an effective method for adoption, has many benefits for shelters, and can tap into a new adopter market by engaging their community in a new way.

## Introduction

Each year, it is estimated that millions of dogs enter the sheltering system in the US [1], [2]. Of those, about 5 to 17 million [3], [4] or as many as 60% do not leave alive [3]. More recent numbers estimate only one quarter of a shelter population finds their way to adoption [5], [6]. Obtaining national statistics on shelter intake and euthanasia is difficult as no central agency or database for the country exists [1]. While trends are improving for shelter dogs with programs for reducing intake, increasing returns to owner, transport, and adoption [7], the chance of a dog dying in a shelter is still high [3].

Every shelter has a maximum number of animals they can care for humanely. This number is based on the number of kennels, trained staff and volunteers to support the animals, services and programs, total live release rate, and length of stay [8], [9]. Minimizing length of stay (LOS) is critical for the minimum care and well-being of dogs in the shelter environment. As LOS increases, then adoptions must also increase, or the average daily population of animals for the staff to care for also increases [9]. Delays must be minimized from intake to adoption in order to maximize adoptions, animal welfare, and reduce crowding. Programs that enable shelters to better manage their kennel space have the potential to increase live release.

Recent American Pet Products Association (APPA) data [10] finds that while 47% of the US population owns a dog, only 20% were acquired from a shelter. Two recent studies show an interesting disconnect between where people consider acquiring their dog and where they actually do. American Humane Association (AHA) [11] reported that more than half (56%) of people who would consider acquiring another dog in their household said they would most likely obtain a dog from a shelter or rescue, a much higher rate than the 22% of people who actually obtained their dog from a shelter or rescue. Garrison (unpublished data) found that when people who acquired a dog in the last year were asked what sources they considered to obtain that dog, over 60% reported they considered obtaining their dog from a shelter. However, of those asked where they actually obtained their last dog, only 39% reported that they obtained their dog from a shelter. This difference between their desire to adopt and their actual behavior of adopting indicates a potential to market shelter dogs better and to increase the dog's visibility to the public to capture more potential adopters.

Given that the majority of people do not acquire their dog from a shelter, and considering that efficient management of kennel space in a shelter can save lives, this research focused on a novel adoption program in which shelter dogs were quickly transferred from the shelter into foster homes. These foster homes, called Adoption Ambassadors, were tasked with marketing the dog and finding an adopter. This program put adoptable shelter dogs in the path of a potential adopter, bypassing the need for the adopter to go to the shelter to adopt. This program, like other foster programs, minimized the staff time, daily care costs, and shelter housing needed [12].

The overall objective of this study was to determine what factors were different between the adopters who adopted from a foster home verses the shelter. Based on a pilot study, it was expected that the adopters would first learn about the dogs in different ways and that those who adopted from an AA foster home would live further away from the shelter. Another expected difference was a shorter length of stay for dogs that were adopted from the shelter compared to those adopted from the foster homes.

## Methods

### LA/SPCA Pilot Study Site

The pilot study was conducted at Louisiana Society for Prevention and Cruelty to Animals (LA/SPCA) in New Orleans, Louisiana. LA/SPCA is a 501(c)3 organization with an open admission policy and contracts to provide animal sheltering and control services to Orleans Parish. The dog population consisted of owner surrendered dogs as well as strays. This study collected data from December 2010 to June 2011 and was developed by the shelter as part of a greater initiative to increase adoptions. LA/SPCA had a total intake of 4692 dogs in 2011. Dogs were assigned into one of two groups based on the last digit of their intake number. If the intake number ended with a 3 or 4, the dog went into the Adoption Ambassador (AA) group and placed into foster homes where the foster ambassador found the adopter. If the last digit of their intake number ended with an 8 or a 9, the dog went into the In-Shelter (IS) group and placed into the adoption area of the shelter, following standard procedure at the LA/SPCA.

Photos were taken of all dogs with a DSLR camera and placed on the shelter's website in the adoption section. Photos and descriptions of each AA dog were emailed to all potential foster homes. Each home could select the dog they wanted to foster on a first-come basis. If the AA dog was not placed into a foster home by Day 3, they were dropped from the study and placed into the shelter adoption area. Moving the dogs into adoption after this time was an important aspect of the program, as it minimized the time that dogs were housed in the holding area where they could not be seen by potential adopters. Length of stay was calculated for both groups from intake to adoption.

After adoption from either group, a survey was emailed to the adopters to gather general information about their previous pet ownership and specific questions pertaining to the recent adoption. After two attempts to reach the adopter by email, a call was made to try to obtain the answers to the survey. Since the sample size of usable surveys was very small, and that these adoptions occurred while the shelter made other changes to increase adoptions, both survey results and LOS data are not included in this manuscript. However, the survey was modified and used at the primary study site. Data were collected on the number dogs returned to the shelter during the study period and the distribution of where the adopters lived. Addresses were obtained by the adoption application completed by the adopter.

### CAS Study Site

The primary study was conducted at Charleston Animal Society (CAS), Charleston, South Carolina. CAS is a 501(c)3 organization with an open admission policy and contracts to provide animal sheltering services to Charleston County. The dog population consisted of owner surrendered dogs as well as strays. CAS was selected as the study site due to the population size of the city, the shelter's prior engagement with community, and the ongoing work with ASPCA. Dogs were included in this study from May through Dec 2012. CAS had a total intake of 3,697 dogs in 2012.

Dogs who were admitted as a stray were housed in a holding area for 5 days and could be reclaimed by their owners. Dogs relinquished by their owner did not need to be held for any duration. All dogs in the shelter were assessed for health, behavior and spayed or neutered before moving into adoption. The selection of dogs for this study occurred on surgery day to ensure all dogs selected were healthy and ready for adoption. If the dog's last digit of their intake number ended with a 3 or 7, the dog went into the AA group and placed into foster homes. If the last digit of their intake number ended with a 2 or a 6, the dog went into the IS group and placed into the adoption area of the shelter. The dogs included all ages, sex, sizes, breeds, and breed mixes. Dogs were not admitted into the study if they were less than 8 weeks of age and were removed from the study if they became sick before adoption.

Like the pilot study, dogs were photographed and moved through the AA and IS groups in the same way. Both groups of dogs were listed on the shelter's website. There were 84 foster homes secured for the AA group during the study period. The AA group had the same supplies and their own local community list for dog-friendly areas. Adopter addresses were collected off the adoption contract at CAS and returns were tracked through August 6, 2013.

### CAS Adopter Surveys

Adopters in both the IS and AA groups were given identical paper surveys during the adoption process. The survey had 10 questions that were a mix of multiple-choice, yes/no, and open-ended formats. The questions consisted of gathering the adopter's previous history of visiting shelters, or adopting animals, how they learned about the current dog being available for adoption and their first impressions of the dog. Also asked was where they first saw the dog, where the adoption occurred, how long it took to decide before adopting, how many pets they had, and sources of their previous pets. The survey responses were manually entered into a Microsoft Excel spreadsheet. The adopters gave written consent that allowed the authors to contact them in the future if needed. No adopters were contacted by the authors. The survey is included with this manuscript as File S1.

The same survey used at LA/SPCA was used for the first 43 adopters with a modification of one question. Instead of being asked “Where did your current pets come from” they were instead asked “Where did all your previous pets came from” in order to capture a more broad experience. In addition, given a low response rate on four additional questions, the wording was modified slightly on those questions for the remaining 104 adopters.

### Adoption Ambassador Group

AA's were volunteers who cared for the dog in their home, found an adopter for the dog, and performed the adoption. Fosters were solicited during the monthly volunteer orientation, flyers in the lobby of the shelter, and through the shelter's marketing and social media outlets.

A coordinator at the shelter was responsible for building the ambassador program and training new recruits. Training for the ambassadors occurred in small groups and some shadowed an adoption that was performed in-shelter. The adoption training, criteria and process were the same for both groups of dogs. Once the ambassadors were comfortable with the process, they would obtain their dog from the shelter along with necessary supplies. Supplies included the adoption application, rabies tag, crate, dog food, collar, leash, business cards with the dog's picture, a list of dog-friendly places to visit in the community, and a brightly colored “Adopt-Me” vest.

The ambassadors used a variety of methods to find an adopter. When they were in public, the dog wore the adoption vest at all times. The ambassadors carried business cards with the dog's photo and their contact information to share with any potential adopters. They notified friends and family and used social media to promote their dog. The adoptions occurred in public places or sometimes in the home of the ambassador. Once the adoption was complete, the ambassador returned the adoption paperwork and adoption fee to the shelter. If the dog was not adopted within a month, the ambassadors were given the choice to return the dog to the shelter. In those cases, the dog was dropped from this study and they would be placed in the shelter adoption area.

### In-Shelter Group

The study did not make any changes to the IS group as this was the typical pathway a dog would flow through the shelter to be adopted. After surgery, dogs were moved to the adoption area as soon as there was an open kennel. The shelter had several fee-waived events and any IS dog in this study who was adopted during one of those events was excluded from the study.

### Statistical Analysis

Geographic Information Systems (GIS) was used to create box plot and 5-mile density maps from the adopter's addresses in both communities. Only records geocoded to a rooftop location or to a narrow street range were left in for the mapping and analysis. There are some limitations to aggregating points into geographic units rather than using natural boundaries. Often these unit boundaries are somewhat arbitrary in terms of the analysis being undertaken and the sizes of the units may change the patterns in the data. With this in mind, the point density surfaces have been created without some of these constraints. These density maps identify high concentrations and clusters of where adopters live, based on the individual addresses and display them in a similar fashion to precipitation maps where more intense color indicates more rain.

**Figure 1:**
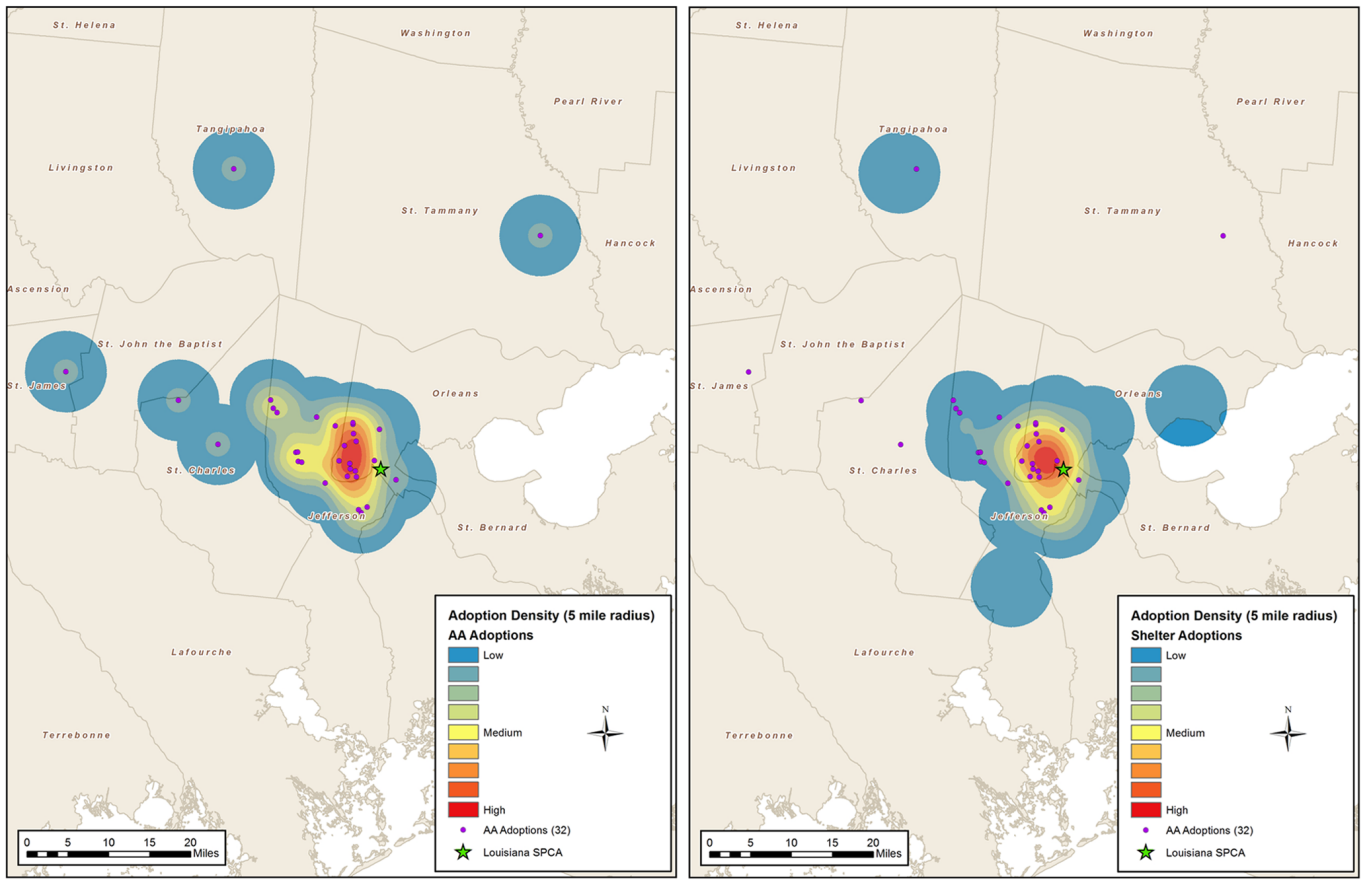
Density maps of adopter addresses in New Orleans, LA.

**Figure 2:**
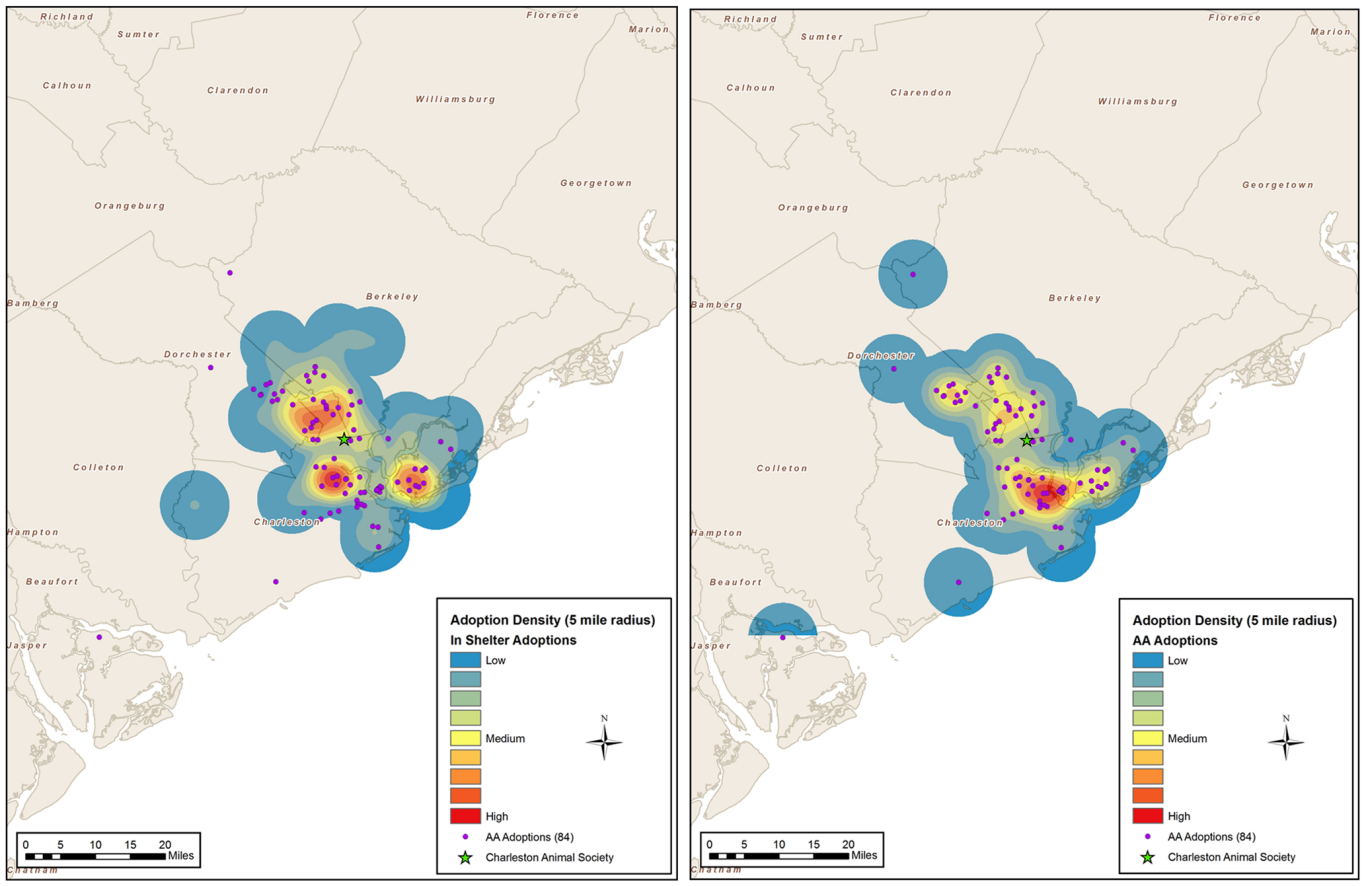
Density maps of adopter addresses in Charleston, SC.

Independent samples t-tests were conducted to compare the distances from the adopter's address to the shelter location between the two groups for each of the two communities (Figures 1 and 2). Mann-Whitney *U* tests were used to compare differences in the medians of the distance from the adopter's address to the shelter location between the IS and AA groups.

For the analysis of the survey questions at CAS, yes/no and multiple choice questions were summarized using frequencies and percentages. For the purposes of analysis, the respondent's written answers to the three open-ended questions were categorized. In order to examine potential differences between the AA and IS groups, Chi-square analysis was used. The Fisher Exact test was used if not enough cells had the expected frequency of 5.

At CAS, LOS was calculated over four time frames: 1. From intake to group assignment, 2. From assignment to available for adoption (either placed in the foster home or placed in adoption at the shelter), 3. From available for adoption to the actual adoption date, and 4. The overall LOS from intake to adoption (total time). Independent samples t-tests were conducted to compare length of stay between the AA and IS groups. Standard statistical software was used for all analysis (StataSE 12, StataCorp LP, College Station, TX, USA).

### Ethics Statement

All adopters were informed verbally that the dog they selected to adopt was part of a research study then asked by shelter staff to provide written confirmation of informed consent. The adopter's addresses were already being obtained from normal operating procedure by the shelter staff. The survey given did not alter the normal procedure of the adoption process at the shelter. Declining to participate in the study did not prohibit the adoption, however no one declined, so no dogs needed to be removed for lack of adopter participation. The focus of this study was on the manipulation of where shelter dogs were adopted and the adopters could elect out of participation, therefore, an ethics committee was not consulted by the authors. Additionally, the data used from the survey were presented as anonymous and complied so individuals are not identified.

## Results

### Returns at LA/SPCA

Returns are those dogs who were adopted into a home and the adopter returned the dog to the shelter within 30 days from adoption. During the study period at LA/SPCA, there were 45 dogs in the AA group and one dog (2%) was returned. There were 55 dogs in the IS group, and six dogs (13%) were returned.

### Returns at CAS

During the study period at CAS there were 84 dogs in the AA group and six dogs (7%) were returned. Out of the 64 dogs in the IS group, 11 dogs (17%) were returned. The authors followed-up with the shelter on Aug 6, 2013 regarding any dogs from this study that were returned beyond thirty days from adoption. There were only four additional returns in the AA group and two additional returns in the IS group. The duration of how long they stayed in their homes after adoption ranged from 1 day to 5 months. Reasons most often cited by the owner for returning the dog were unrealistic expectations and resident dog not getting along with newly adopted dog. One person reported being allergic and one person had an issue with their landlord.

### GIS Mapping of LA/SPCA and CAS Adopters

Distances from the adopter's address and the shelter were calculated using mileage. Outliers that were 2 standard deviations out were not included in the analysis. Table 1 summarizes the mean, standard deviation, and median for the two communities, New Orleans (NO) and Charleston (CH), and the two groups (AA and IS) in each community.

**Table 1:** Descriptive statistics for the two groups (AA and IS) in for the two communities.

	New Orleans		Charleston	
	IS	AA	IS	AA
Observations	43	33	61	85
Mean	6.16	12.45	11.08	11.83
Standard Deviation	6.61	13.68	18.57	19.92
Median	5.12	6.56	8.24	9.23

In New Orleans, the average distance from the shelter for the AA group was significantly longer than that of the IS group (t(74) = 2.644, p = .010). The median distance for the AA group in NO was also significantly longer (z = 2.657, p = .008). Neither the average distance nor the median distance were significantly different for IS and AA in Charleston.

All addresses from both communities were compiled to create 5-mile density grid maps that aggregated points into geographic units. In New Orleans, a total of 75 addresses (32 AA, 43 IS) were used for mapping (Figure 1). In Charleston, a total of 144 addresses (84 AA, 60 IS) were used in the final analysis for density mapping (Figure 2). The density maps show where the adopters lived with a darker color representing more adopters.

### Length of Stay at CAS

A total of 203 dogs were selected for this research and resulted in 103 dogs in the AA group and 100 in the IS group. For the purposes of evaluating length of stay (LOS), 19 dogs were removed from the AA group. Exclusion was based on: four could not stay in their foster homes due to outside circumstances (such as vacation), four were not placed into foster by the third day after selection and were placed on the adoption floor, two had behavioral concerns with the resident dog; one was required to be adopted with his previous housemate; and two developed a skin condition where they needed lengthy treatment in the foster home. An additional 6 dogs were returned to the shelter and those were excluded. A total of 36 dogs were excluded from the IS group because 25 were adopted during a fee-waived event at the shelter and another 11 were returned.

After these exclusions, there were a total of 148 dogs; AA had 84 dogs and IS had 64 dogs. The AA group had 50 female dogs, 34 male dogs and an average age of 1.7 years. The IS group had 38 female dogs, 26 male dogs and had an average age of 1.1 years. Both groups had the same median age of 0.8 years.

The AA group was significantly different than the IS group in three of the four LOS time conditions. See Table 2 for median, max and standard deviations for the adopted dogs in the two groups. Overall, the AA group took significantly longer than the IS group to move through the entire process of intake to adoption (t(146) = 5.935, p<.001). The AA group had a significantly longer LOS from the time they were made available for adoption to the time of the actual adoption (t(146) = 6.786, p<.001). Once assigned to a group, the IS dogs took significantly longer to move to the adoption area than the AA dogs took to go into a foster home (t(146) = −5.613, p<.001). From intake to selection into a group, the AA dogs had slightly longer LOS before their surgery, however it was not significant.

**Table 2:** Descriptive statistics for length of stay for each time period for each group.

	Intake to Assignment		Assignment to Available		Available to Adoption		Overall Intake to Adoption	
	AA	IS	AA	IS	AA	IS	AA	IS
mean	6.79	5.63	1.26	3.75	15.73	2.58	24.77	11.95
median	6.00	4.00	1.00	2.50	9.00	1.00	20.50	10.00
max	23.00	33.0	8.00	14.00	75.00	14.00	82.00	35.00
sd	4.25	5.77	1.51	3.68	15.24	3.13	16.18	6.92

### Original vs. Revised Survey analysis at CAS

Of the 148 eligible dogs, 147 adopters completed a survey with sufficient information for analysis. The original survey was completed by 43 adopters (21 AA, 22 IS) and 104 completed the revised survey (53 AA, 51 IS). Only four questions were different between the two surveys.

For the two questions that differed slightly in wording, one was a yes/no question (“I was already considering adding a dog to our family before seeing this dog”) and the other was an open ended question (“I first learned about CAS by”). As described, the responses to the open-ended questions were categorized for analysis. Chi square analysis was used to compare the answers to these two original and revised questions and there were no significant differences found. One open ended question was changed to a seven-choice multiple choice question (“I have not adopted an animal from a shelter before because”) because only 10 responded to this follow-up question in the original survey. This question still provided an “Other” open field. The fourth question (“I first learned about this dog being available for adoption”) had one choice added to the other multiple choice options.

The surveys were combined because two of the questions showed no significant differences in responses and the other two questions with added choices included an open field that resulted in a variety of responses. Surveys were not included from the 9 AA homes that adopted their AA foster dog.

### Combined Survey Results at CAS

The majority of respondents to the survey were already considering adding a dog to their family (93%, n = 136), had visited an animal shelter before (86%, n = 120), and had visited CAS in the past (63%, n = 82). The most common ways adopters learned about CAS were through internet sources such as website, Facebook, or Petfinder (37%, n = 50), through someone they knew (29%, n = 40) and media such as TV, newspaper, radio or the phone book (15%, n = 21). Fifty-four percent (n = 78) had not adopted from a shelter or rescue before. Respondents who had not adopted before were asked what prevented them from adopting from an animal shelter. Of the 64 people who responded to this question, the top three reasons given were no opportunity/never visited (38%, n = 24); wanted a particular breed (22%, n = 14) or they acquired the pet somewhere else or already had pets (9%, n = 6). Most people who answered the question (n = 102) decided to adopt after one visit (77%, n = 78), some needed two or more visits (17%, n = 17), and a few didn't need any visits (7%, n = 7). There were no significant differences between the AA and IS groups in any of these areas.

**Figure 3:**
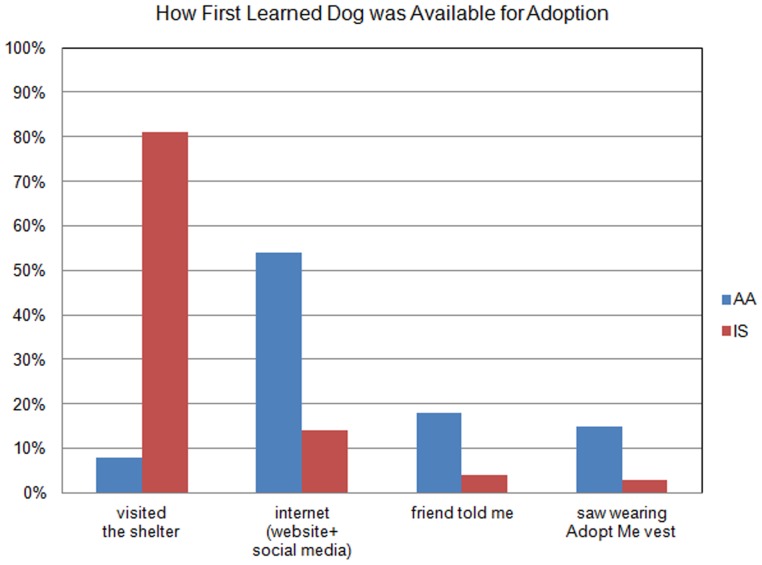
How adopters first learned dog was available for adoption.

There were significant differences in how the groups first learned about the dog available for adoption (*X* ^2^(3) = 73.6, p<.001) as seen in Figure 3. The majority of the IS dogs were visited at the shelter (81%, n = 59). The AA adopters saw the dog on internet sources (54%, n = 40), were told about the dog by a friend (18%, n = 13), or saw the dog out in public wearing the Adopt-Me vest (15%, n = 11).

As expected, there were significant differences in the location of adoption. The majority of IS dogs were adopted at CAS (92%, n = 66) and the majority of AA dogs were adopted directly from the ambassador (97%, n = 71). Also significantly different was the time it took to decide to adopt. The vast majority of the IS group (93%, n = 66) decided in a few hours or less, while 78% of the AA group (n = 54) did. Nearly 30% of the AA group took one day or more compared to only 10% of the IS group (*X* ^2^(1) = 6.34, p = .012).

The responses for the open ended question “My first impressions of this dog were” were grouped into 9 categories plus “Other.” Appearance was mentioned most often for both groups (58%, n = 78), as well as behavior with people (33%, n = 44). When asked where they acquired their previous dogs, the sources chosen by adopters where fairly evenly split among the top three categories (see Table 3).

**Table 3:** Sources where adopters acquired their previous dogs.

	AA		IS		Total	
	%	N	%	N	%	N
**shelter/rescue**	29	18	30	19	30	37
**breeder**	27	17	24	15	26	32
**family/friend/neighbor**	19	12	32	20	26	32
**Pet store**	5	3	6	4	6	7
**found as stray**	8	5	2	1	5	6

**Figure 4:**
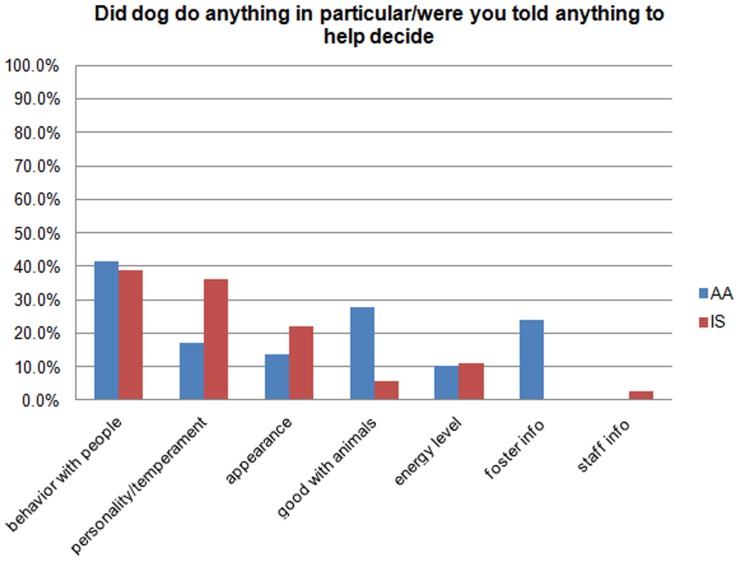
Did the dog do anything in particular/were you told anything to help decide.

When asked if the dog did anything in particular, or if they were told anything specific about the dog that helped them make a decision, behavior with people was the most common response (41%, n = 26) among the people who provided a response beyond “No” (Figure 4). While personality/temperament was second most reported by the IS group (36%, n = 13), being good with animals was second for the AA group (28%, n = 8). Information from the foster home was reported from 24% (n = 7) in the AA group, while information from staff was reported from 3% (n = 3) in the IS group.

## Discussion

This study examined the effectiveness of a novel adoption program in which foster homes were tasked with not only housing the pet, but in finding the new adoptive home for the pet. Through the examination of dogs adopted through traditional methods and dogs adopted through the novel adoption ambassador program, we found that the novel approach can be an effective method for adoption. One key difference between groups in both communities was a lower return rate for the dogs that were adopted through the AA program compared to the IS group. Follow-up in Charleston was for a longer duration and returns stayed low. Decreasing returns results in using fewer shelter resources, including kennel space, and thus increases the potential to save more lives.

One possible reason for lower returns in the AA group may be that adopters are able to make a more informed decision about the pet's behavior in the home. Nearly a quarter of AA adopters who responded to the question reported that information they received from the foster ambassador helped them decide to adopt, while only 3% of people reported that information they received from staff about an IS dog helped them decide. As the foster ambassadors lived with the dogs in their home, they were likely to be able to impart more relevant information to the adopter. The difference may also be due to adopters see the AA dog in a place they visit during their normal day, they may be able to better envision what life might be like with that dog. Research shows that when adopters have realistic expectations, they are more likely to keep their pet [13] and can be key in avoiding relinquishment in the future [14].

Length of stay in the shelter can be an impactful driver for increasing live release rate and in enhancing the care pets receive [8], [9]. At CAS, the IS dogs took significantly longer to move into adoption after sterilization than the AA dogs took to be picked up by their foster homes. Even though the IS dogs were ready for adoption, they were housed away from public view until a kennel opened in the adoption area. Remaining in the holding area created a bottleneck for the IS dogs that resulted, on average, in a nearly 4 day wait for an open kennel in adoptions. The AA program was designed to have enough foster homes prepared to take the dog out of the shelter right after surgery and on average; the AA dogs were available for adoption after one day. Ensuring foster homes were prepared to take a dog, resulted in a shorter shelter stay for each AA dog.

The AA program also freed up kennel space and possibly allowed for better kennel space management for IS dogs. Post hoc, we looked at LOS of dogs at CAS for the year prior to this study. In the year 2011, LOS for dogs in CAS was 15 days. During our study period, LOS for the IS dogs was only 12 days. Although different adoption programs and other policy differences were in effect compared to the time frame of our study, it is feasible that the AA program did have an effect since on average during the study there was a 4% decrease (approximately 12 dogs per month) in the number of dogs housed in-shelter because they were in the AA foster homes. Decreasing LOS reduces the average daily population of animals that staff needs to tend to and can have tremendous impact on quality of care [9].

Once in the AA home, the overall LOS was longer to adoption, but this was not detrimental given the dog was free from the stress levels of being in the shelter [15] and had benefits of being in the foster home [11]. The foster home also most likely offers more frequent interaction which can improve the dog's behavior and increase their chance of being adopted [16], [17]. Dogs can easily transfer their learning to new people and places [18] so while in foster care they can be taught life skills, such as waiting at a door or recalls, that will set them up for success for their new home.

As the majority of the pet owning public do not obtain their pet from a shelter [10], finding those that desire pets that may or may not have come to the brick and mortar facility could lead to a bigger share of the pet owning public. In both communities, the adopters that obtained their pets in-shelter (IS) compared to those that adopted through AA differed in their distribution. In New Orleans, the groups where significantly different in distance with the AA group being significantly farther from the shelter. In Charleston, the distances were not significantly different, but those who adopted from the AA group had one core area and those who adopted from the IS group had three distinct areas, with only one of them having overlap with the AA adopters, indicating that the adopters were concentrated in different areas. By taking the dogs to where the adopters spend their time, dogs were adopted from people who lived in a different part of town then those adopting from the shelter. As noted in the results, only 29% of the AA adopters who responded to the question said that any of their previous pets had come from a shelter or rescue. Since 93% of the adopters reported they were already considering a dog, there is the potential that the AA dog captured someone who would have obtained their dog from one of the other resources such as breeder and pet store.

The AA program can be implemented with little cost to the shelter. The program requires only a few additional supplies above those normally provided to a foster home, namely an adoption vest and a business card for each dog. The vests can be made by volunteers or purchased on-line for a nominal fee. The business cards can be made by either the foster home or the shelter using a computer and standard printer paper. The shelter needs to have a staff member who can manage the program, which would generally be the foster coordinator or someone who oversees the adoption process.

Limitations of this study include lack of follow-up in the home with the adopters to know if they kept the dog or re-homed on their own. The density maps are made on a 5-mile grid which has limited applications. A map that used the city neighborhoods or natural boundaries would create a better application of the dataset. A larger dataset in New Orleans with the more thorough updated survey given at the time of adoption would provide direct comparisons between the two communities. There were over 80 AA homes in Charleston and many fostered several dogs over the duration of this program. However, each home was not tracked individually to determine the number of adoptions from each home. Also, although the authors randomly assigned which dogs would go to the AA program, the foster homes selected the dog based on photo and description. The authors accepted this bias as it is realistic and functional for program; the ambassadors were encouraged to take a dog they can adopt in order to have a positive experience and continue in the program.

In conclusion, those who adopted the AA dogs lived in different parts of the community than those who adopted from the shelter. Although the AA dogs have a longer length of stay from intake to adoption, they have a shorter stay in the shelter and allow more kennel space in adoption for other dogs in-shelter. The public learned about dogs being available in the AA homes through social media, hearing about the dog from a friend, and seeing the dog wearing the adoption vest. Dogs adopted from the AA group had few returns in both communities. There were fewer differences than expected between the two groups of adopters. This program has many benefits for shelters and can tap into a new adopter market by engaging their community in a new way.

## Supporting Information

File S1 **Adopter Survey.** This survey was completed by all adopters in both groups (AA and IS) during the adoption process.
(PDF)
