# Supplementary material for: Evaluation of a Novel Dog Adoption Program in Two US Communities
Source: PLoS One. 2014 Mar 24;9(3):e91959. doi: 10.1371/journal.pone.0091959 (PMC3963870; doi:10.1371/journal.pone.0091959)
Supplement: File S1 — Adopter Survey. This survey was completed by all adopters in both groups (AA and IS) during the adoption process. (PDF) [file pone.0091959.s001.pdf]

Charleston Animal Society (CAS)
Adoption Survey

Date: ____/____/ 2012 Dog's name given by shelter:______________________

1. My (Adopter) Info:
   Name (name on the Adoption contract): _______________________________
   Address: _________________________________City, State: ________________ Zip:_____

2. I first learned about Charleston Animal Society (CAS) by ________________________

3. In the past, I have visited: an animal shelter *(circle one)* Yes/No
   Charleston Animal Shelter *(circle one)* Yes/No

4. Choose one that applies:
   - ❑ I *have* adopted an animal from a shelter or rescue group before. My most recent adoption before today was a *(circle one)* dog/ cat / other on: Month ___________ Year _______
   - ❑ I *have not* adopted an animal from a shelter or rescue group before because (*Check all that apply):*
     - ❑No opportunity/never visited
     - ❑Wanted a particular breed
     - ❑Thought I would feel sad when visiting the shelter
     - ❑Too many animals to choose from
     - ❑Location of shelter not convenient for me
     - ❑Did not think shelter would have an animal I liked
     - ❑Didn't want an animal, then be denied for some reason
     - ❑Concerned that shelter animals might have behavior/medical issues
     - ❑Haven't had a pet before
     - ❑Other: ____

5. I first learned about this dog being available for adoption (*Check all that apply):*
   - ❑On the shelter website
   - ❑Via social media website (ex: Facebook)
   - ❑Calling the shelter
   - ❑Visiting the shelter
   - ❑ From a friend
   - ❑ While at an off-site adoption event at: ______________________________
   - ❑Saw the dog in a public place wearing an "Adopt Me" vest. *Where were you When you first saw your dog?)*_____________________________________
   - ❑Other (please describe):

(Please continue to Page 2)

6. I was already considering adding a dog to our family before seeing this dog. *Yes / No*

7. My first impressions of this dog were (*i.e., what caught my eye?)*__________________
____

8. Did this dog *do* anything particular or were you *told* anything specific about him/her that helped you decide to adopt this dog? If so, please describe: ____________________________
____

9. I am adopting this dog:
- ❑Directly from the foster guardian (*in their home or at public place)*
- ❑From an off-site adoption event
- ❑At the Charleston Animal Society shelter
- ❑Other : ______________________________

10. I visited with this dog ______times before deciding to adopt him/her and decided when:
- ❑As soon as I saw him/her, I wanted to adopt
- ❑I needed up to an hour of "meet and greet"
- ❑I needed a day to think about it
- ❑I needed a couple of days (or more) to decide
- ❑Other ________________________________

11. Not counting the dog I am adopting now, I currently have the following pets:

Dogs 0 1 2 3 4 5 6 7 8+

Cats 0 1 2 3 4 5 6 7 8+

Other _________________________

12. My previous pets have come from (*Check all that apply:*

	Shelter/ Rescue	Breeder	Friends, family, neighbors	Pet store/ Market	Found as stray	Craigslist/ Newspaper
Dogs						
Cats						
Other?						

Comments:

Thank you for your time! Feel free to add any additional comments below:
